# Phylogenetic reconstruction of Tuberolachnini and Lachninae (Insecta, Hemiptera): Morphological and molecular analyses revealed a new tribe

**DOI:** 10.1186/s12983-024-00550-2

**Published:** 2024-11-19

**Authors:** Mariusz Kanturski, Yerim Lee, Hyojoong Kim

**Affiliations:** 1 Institute of Biology, Biotechnology and Environmental Protection, Faculty of Natural Sciences, University of Silesia, Bankowa 9, 40-007 Katowice, Poland; 2 Department of Biological Sciences, Kunsan National University, 558 Daehak-Ro, Naun 2(I)-Dong, Gunsan-Si, Jeollabuk-Do, Republic of Korea

**Keywords:** Lachnini, Morphology, *Prunus*, SEM, Tuberolachnini, Sensilla

## Abstract

**Background:**

Lachninae (Insecta: Aphididae) represent a fascinating group of aphids that are traditionally divided into five tribes. Among these, members of the tribe Tuberolachnini exhibit remarkable morphological and biological diversity. One genus of this group, *Miyalachnus,* known from Japan, is characterized by unique features. Our study aimed to re-examine the tribal classification within Lachninae, with a focus on the diverse Tuberolachnini and the previously understudied genera *Miyalachnus* and *Sinolachnus*.

**Results:**

We conducted a comprehensive phylogenetic analysis using four genes (COI, COII, CytB, and EF1α), employing both maximum likelihood (ML) and Bayesian inference (BI) methods on a combined dataset. Our findings challenge the monophyly of Tuberolachnini. The analyses revealed that *Miyalachnus* and *Sinolachnus* are phylogenetically distinct from the core Tuberolachnini genera (*Nippolachnus*, *Pyrolachnus*, and *Tuberolachnus*), instead showing a closer relationship with Tramini. Specifically, the *Miyalachnus* clade forms a sister clade to the clade containing *Sinolachnus* and Tramini.

**Conclusions:**

On the basis of these molecular results, corroborated by morphological evidence, we propose to erect a new tribe within the Lachninae-Miyalachnini **trib**. **nov**. with *Miyalachnus* as the type genus. We also provide updated taxonomic diagnoses for the remaining tribes and discuss their relationships as well as distinguishing features.

**Supplementary Information:**

The online version contains supplementary material available at 10.1186/s12983-024-00550-2.

## Background

The so-called true aphids (Hemiptera: Aphidomorpha: Aphididae), comprising approximately 5,300 valid species [1], constitute one of the most significant insect groups because of their very high degree of diversity and biology. Because aphids often live in numerous colonies and have enormous environmental plasticity and complex life cycles, aphids have a high degree of polymorphism, which has given them great evolutionary success [2–6]]. For this group, there have been several studies, mostly on their still unsolved molecular phylogeny [7–9] undetermined relationships within subfamilies, tribes or genera [10–19] and investigations into diversity [20–23].

**Fig. 1:**
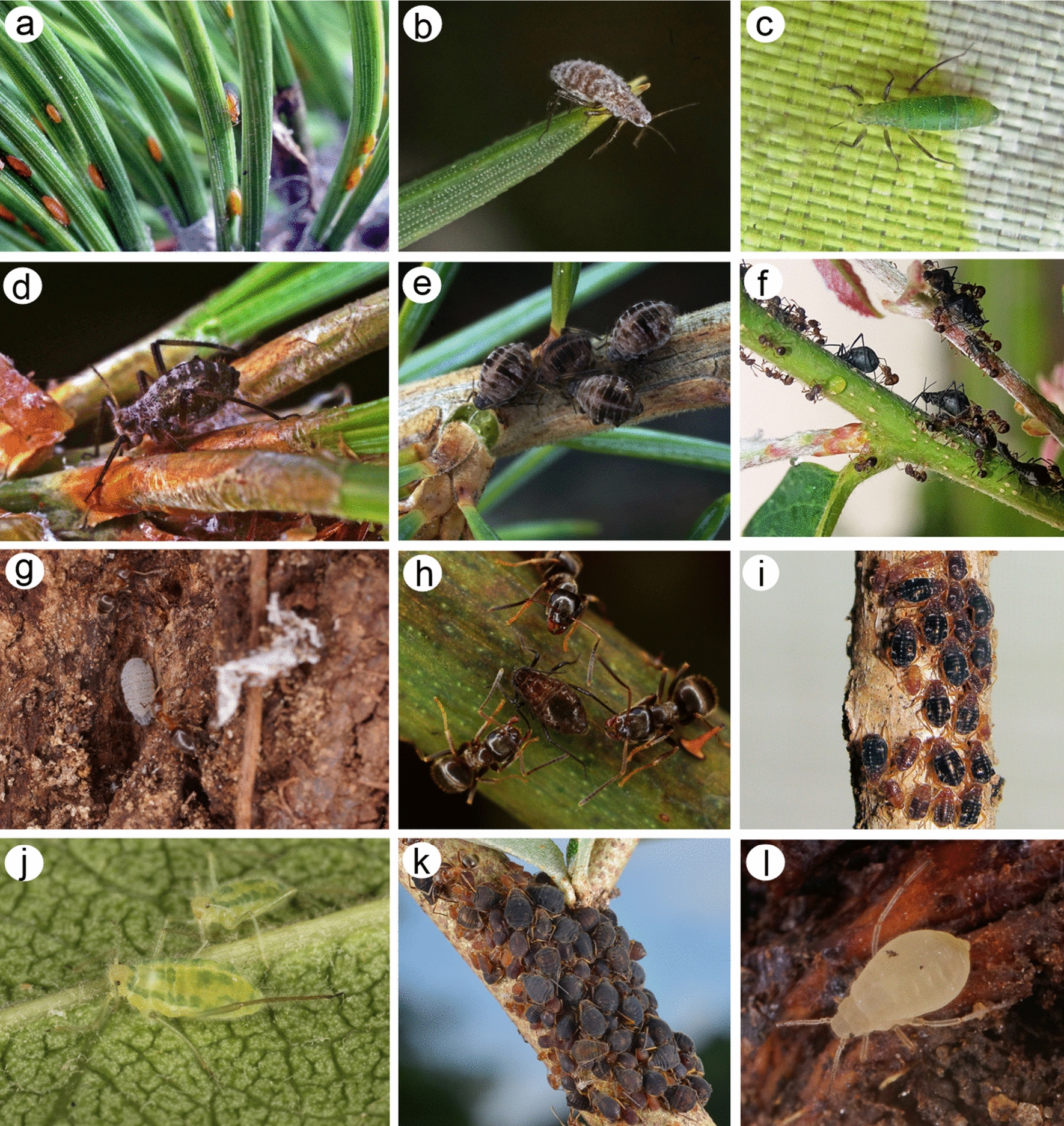
Lachninae representatives in life: (**a**) *Eulachnus brevipilosus*, (**b**) *E. rileyi*, (**c**) *Pseudessigella brachychaeta*-Eulachnini representatives feeding on needles; (**d**) *Cinara pinea*-Eulachnini representative feeding on green shoots; (**e**) *C. cedri*-Eulachnini representative feeding on woody shoots; (**f**) *Lachnus tropicalis*-Lachnini representative feeding on green shoots; (**g**) *Stomaphis longirostris*-Stomaphidini representative feeding on trunks; (**h**) *Maculolachnus submacula*-Lachnini representative feeding on green or woody shoots; (**i**) *Miyalachnus sorini*-Miyalachnini representative feeding on woody parts; (**j**) *Nippolachnus piri*-Tuberolachnini representative feeding on leaves; (**k**) *Sinolachnus yushanensis*-Tramini representative feeding on woody shoots and branches; (**l**) *Trama troglodytes*-Tramini representative feeding on roots

Lachninae Herrich-Schaeffer, 1854, is one of the 23 subfamilies within Aphididae [1, 18] and is characterized by the largest size among aphids, which feed on both the green and woody parts of both coniferous and deciduous plants [24, 25]. A majority of the species are associated with trees and shrubs, with three root-feeding genera from the tribe Tramini Herrich-Schaeffer, 1854 [4, 26] (Fig. 1). The classification of Lachninae has undergone several changes over time, particularly at the tribal level. For many years, only three tribes have been recognized, and their classification is based on the biology and ecology of their members: Lachnini Herrich-Schaeffer, 1854 (with species that feed on deciduous trees and shrubs), Cinarini Börner, 1930 or Eulachnini Baker, 1920 (whose representatives are associated with conifers) and Tramini (with species that feed on the roots of deciduous plants) [e.g., 3, 27, 28]. Normark [29] was the first to challenge this classification using molecular analysis. His work suggested that more than three tribes could be distinguished in this group, revealing that some genera formed separate clades for the two tribes, Tuberolachnini Oestlund, 1942 and Stomaphidini Mordvilko, 1914. Finally, the current five-tribe classification of Lachninae was reconfirmed by Chen et al. [11] with molecular phylogenetic evidence and the feeding ecology of numerous genera and species.

### Previous classification and phylogenetic hypotheses of Lachninae

**Fig. 2:**
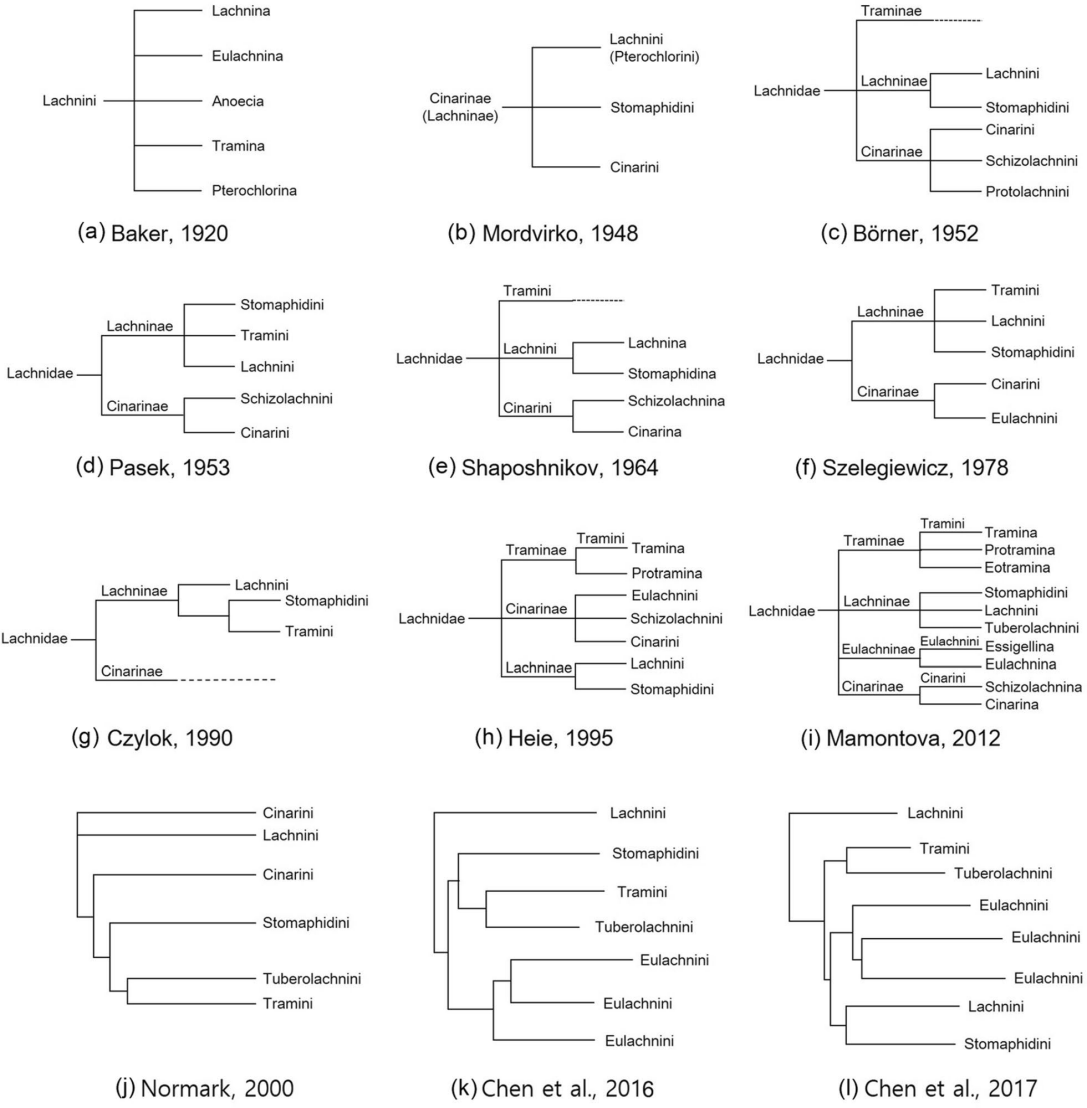
Taxonomical and phylogenetic hypotheses on Lachninae: (**a**) classification of Baker, 1920; (**b**) classification of Mordvilko, 1948; (**c**) classification of Börner, 1952; (**d**) classification of Pašek, 1953; (**e**) classification of Shaposhnikov, 1964; (**f**) classification of Szelegiewicz, 1978; (**g**) phylogenetic classification of Czylok, 1990; (**h**) classification of Heie, 1995; (**i**) classification of Mamontova, 2012; (**j**) molecular phylogeny of Normark, 2000; (**k**) molecular phylogeny of Chen et al., 2016; (**l**) molecular phylogeny of Chen et al., 2017

The classification system of Lachninae has undergone considerable changes over time, with its taxonomic position and composition varying on the basis of different researchers. One of the earliest attempts to determine the relationships of Aphididae was made by Baker [30], who distinguished only one tribe, Lachnini with five subtribes (Fig. 2a). In the hypothesis proposed later by Mordvilko [31], Lachninae (synonymous with Cinarinae Börner, 1930) had a subfamily level with three tribes: Cinarini, Stomaphidini and Lachnini (Fig. 2b). In this classification system *Tuberolachnus* Mordvilko, 1908 together with *Stomaphis* Walker, 1870, *Maculolachnus* Gaumont, 1920, and *Trama* von Heyden, 1837 were grouped into one tribe-Stomaphidini. On the other hand, Börner [32] presented Lachninae at the level of the family (Lachnidae) with three subfamilies: Cinarinae, Lachninae and Traminae Herrich-Schaeffer, 1854 (Fig. 2c). According to this hypothesis, five genera belong to the tribe Lachnini, one of which is *Tuberolachnus*. One year later, Pašek [33] proposed only two subfamilies within Lachnidae (Lachninae and Cinarinae), in which Lachninae was subdivided into three tribes (Fig. 2d). In Pašek’s classification one of the tribes-Lachnini was subdivided into two groups of genera: *Trama*, *Maculolachnus* and *Tuberolachnus* in one group and *Lachnus* and *Schizodryobius* in the other. Shaposhnikov [34] presented his classification with three tribes within Lachnidae and the subtribe Lachnina in Lachnini consisting of *Lachnus*, *Pterochloroides*, *Maculolachnus* and *Tuberolachnus* (Fig. 2e). On the basis of the sometimes unclear decisions of previous authors, the hypothesis presented by Szelegiewicz [35] also took into account the ecology and plant associations of lachnids (Fig. 2f). Lachnidae has been subdivided into two subfamilies: Cinarinae (members that feed on conifers) and Lachninae (which can be found only on deciduous plants). The subfamily Lachninae comprises three tribes classified according to their feeding habits or place: Stomaphidini (with *Stomaphis* on the trunks), Lachnini (with *Tuberolachnus*, *Lachnus* and *Maculolachnus* on branches and shoots) and Tramini, whose members feed on roots. A breakthrough in the classification and phylogenetic relationships of lachnids took place when Czylok [36] presented his phylogenetic concept, which focused mainly on Tramini and some other lachnids on the basis of cladistic analysis results. Czylok [36] subdivided Lachnidae into Lachninae and Cinarinae. Lachninae contains three tribes: Lachnini (with *Longistigma* Wilson, 1909; *Lachnus*, *Tuberolachnus*, *Maculolachnus*, *Nippolachnus* Matsumura, 1917, *Pyrolachnus* Basu & Hille Ris Lambers, 1968 and *Sinolachnus* Hille Ris Lambers, 1956), Stomaphidini and Tramini (Fig. 2g), among which a clade of the latter is sister to the former one. This hypothesis has not been widely accepted unfortunately, and sometime later Heie (1995) presented his own classification system for Lachnidae with three subfamilies (Fig. 2h), which was very similar to the proposal of Szelegiewicz (1978). In the Heie [24] classification, *Tuberolachnus* was also a member of Lachnini within Lachninae. The last and indeed most controversial classification system, which diverges considerably from those already presented, is the system proposed by Mamontova [37]. In her hypothesis, Mamontova [37] treated Lachninae at the family level and distinguished four subfamilies (Cinarinae, Eulachninae Baker, 1920; Lachninae and Traminae) (Fig. 2i). Within the Cinarinae, we can find one tribe, Cinarini, subdivided into two subtribes: Schizolachnina Börner, 1949 (with *Schizolachnus*), and Cinarina Börner, 1930, which contains *Cinara* and eight genera, the names of which are currently treated as subgenera or synonyms of *Cinara* (*Cinarella* Hille Ris Lambers, 1948; *Cinarellia* Börner, 1952; *Buchneria* Börner, 1952; *Cupressobium* Börner, 1940; *Todolachnus* Matsumura, 1917; *Lachniella* Del Guercio, 1909; *Cinaropsis* Börner, 1939 and *Cedrobium* Remaudière, 1954). The subfamily Eulachninae has one tribe, Eulachnini, which is subdivided into Eulachnina (with *Eulachnus*) and Essigellina Mamontova, 2008, for *Essigella* del Guercio, 1909; *Pseudessigella* Hille Ris Lambers, 1966; *Archeoessigella* Sorensen, 1994; and *Lambersella* Sorensen, 1994 (the latter two were later synonymized by Théry et al. [38] with *Essigella*). The subfamily Traminae also has one tribe, Tramini, which is subdivided into three subtribes: Eotramina Czylok, 1990; Protramina Eastop, 1953; and Tramina. Finally, the subfamily Lachninae is subdivided into three tribes: Tuberolachnini for *Tuberolachnus*, Stomaphidini for *Stomaphis* and *Maculolachnus*, and Lachnini. Notably, in the tribe Lachnini, Mamontova [37] included ten genera: *Lachnus*, *Longistigma*, *Schizodryobius* van der Goot, 1913 (synonym of *Lachnus*), *Sublachnobius* Heinze, 1962 (synonym of *Lachnus*), *Pterochloroides*, *Linolachnus* Hille Ris Lambers (*sic*), *Pyrolachnus*, *Nippolachnus*, *Neonippolachnus* Shinji, 1924 and *Sinolachnus*. This controversial system has also been upheld by Mamontova [39, 40], with two changes*—Nippolachnus* has been transferred to Tuberolachnini and *Neonippolachnus* has been excluded from the classification.

### Current classification and phylogenetic relationships of Lachninae

The modern classification of Lachninae began with Normark’s [29] publication of the first molecular phylogeny of the subfamily. Prior to this, classifications were based on the previously proposed theories, mostly those of Heie [24], which drew partly from Szelegiewicz [35] but downgraded the family level to Lachninae. In 1997, Remaudière & Remaudière’s *Catalogue des Aphididae du Monde* (Catalogue of the world’s Aphididae) [27] treated Lachninae as a subfamily with three tribes: Cinarini (*Cinara*, *Eulachnus*, *Essigella*, *Pseudessigella*), Lachnini (*Lachnus*, *Longistigma*, *Maculolachnus*, *Tuberolachnus*, *Neonippolachnus*, *Nippolachnus*, *Pterochloroides*, *Pyrolachnus*, *Sinolachnus*, *Stomaphis*) and Tramini (*Eotrama* Hille Ris Lambers, 1969; *Protrama*, *Trama*). This classification persisted even after Normark’s [29] findings, which suggested the existence of five tribes within Lachninae. Normark proposed restoring two additional tribes: Stomaphidini (for *Stomaphis*) and Tuberolachnini (including *Tuberolachnus* and *Nippolachnus)* (Fig. 2J).

A new era in Lachninae classification and phylogenetic relationships began with Chen et al. [11]. They confirmed the five-tribe classification of the subfamily (Fig. 2k) and provided new data, including ancestral reconstruction. A similar model of relationships was presented by Chen et al. [41] using *Buchnera* (Fig. 2l).

The most recent change in Lachninae systematics was proposed by Kanturski et al. [19]. They provided morphological evidence for species of the genus *Sinolachnus* (treated as a member of Tuberolachnini), which was transferred to the tribe Tramini because they share many more similar features with *Eotrama* than with the remaining Tuberolachnini.

### Tuberolachnini system history

Tuberolachnini stands out as one of the most diverse tribes within Lachninae. Members of this group exhibit considerable variation in their morphology, host plant range and feeding location. The key characteristics of tuberolachnines include being rather densely covered in long, fine, pointed setae, with the first segments of tarsi having 1–1–1 sense pegs (*Indolachnus* and *Nippolachnus*) or more than one (e.g. *Miyalachnus*, *Pyrolachnus* and *Tuberolachnus*). Species of the genera *Indolachnus* and *Nippolachnus* are the most distinctive in their morphology owing to their pear-schaped (*Indolachnus*) and narrow (*Nippolachnus*), delicate body and feeding place—the leaves and petioles of woody Rosaceae (e.g., *Eriobotrya*, *Pyrus*, *Rhaphiolepis* and *Sorbus*). *Tuberolachnus* members are large aphids characterized by their dorsal abdominal tubercle and living on the green or woody branches of various species of *Salix* (Salicaceae) or *Eriobotrya* (Rosaceae).

The phylogenetic relationships within Lachninae, particularly in the tribe Tuberolachnini, remain a subject of ongoing debate in aphid taxonomy. Some members of this tribe are still poorly defined, leading to controversies among researchers [3, 4, 19, 23]. Tuberolachnini comprises medium to large aphids that feed on branches or leaves of deciduous trees and shrubs, with a preference for Rosaceae and *Salix* (Salicaceae). Over the years, various studies have shaped our understanding of this group. The tribe’s history dates back to 1942, when Oestlund [42] first used the term for the genus *Tuberolachnus*. As presented in previous paragraphs, the idea of Tuberolachnini as a separate tribe was neither widely accepted nor used. In fact, Mamontova consistently recognized Tuberolachnini as a separate tribe in her publications over several decades, with *Tuberolachnus* as the only member [37, 43, 44] and later also including *Nippolachnus* as the second member [39, 40]. Later, Normark [29] conducted the first molecular phylogenetic analysis of Lachninae, resulting in a new tribal classification that grouped *Nippolachnus* and *Tuberolachnus* together. These findings were robustly confirmed by subsequent studies, especially during molecular analyses [11, 41]. Chen et al.'s [11] study further expanded the tribe by including *Pyrolachnus* and suggested the transfer of *Neonippolachnus* and *Sinolachnus* from Lachnini. However, Chen's study [11] did not include results from morphological analysis or wider discussion. Recent research has challenged some of these classifications. Kanturski et al. [19] proposed that *Sinolachnus* might be more closely related to Tramini than to Tuberolachnini. The following year, Kanturski et al. [45] revised the genus *Nippolachnus*, establishing a new genus, *Indolachnus,* and clarified the status of *Neonippolachnus* as a synonym of *Nippolachnus*. Finally, Kanturski & Lee [46] analysed the identity of *Pyrolachnus imbricatus nipponicus* described by Sorin [47] from Japan and, owing to the significant differences from other *Pyrolachnus* species and genera, proposed a new genus, *Miyalachnus,* within Lachninae.

Given these taxonomic ambiguities, our study aimed to thoroughly investigate the monophyly of Tuberolachnini. We conducted a comprehensive analysis combining detailed morphological examinations with molecular phylogenetic techniques. Notably, our study is the first to include *Miyalachnus* and *Sinolachnus* in phylogenetic testing while also incorporating the largest number of Lachninae species to date. Our goal was to clarify the relationships within this tribe and potentially resolve some of the longstanding uncertainties in its classification. As a result of morphological differences and characters, supported by molecular data, a new tribe, Miyalachnini, is proposed, and it is classified as a sister group of Tramini, in which *Sinolachnus* is a confirmed member.

## Materials and methods

### Study material, light microscopy and abbreviations

Type species of all Lachninae genera used for this research were examined: *Cinara pini*, *Eotrama moerickei*, *Eulachnus agilis*, *Essigella californica*, *Indolachnus himalayensis*, *Lachnus roboris*, *Longistigma caryae*, *Maculolachnus submacula*, *Miyalachnus sorini*, *Nippolachnus piri*, *Protrama radicis*, *Pseudessigella brachychaeta*, *Pyrolachnus pyri*, *Trama troglodytes* and *Tuberolachnus salignus*. The slide-mounted specimens of available representatives of Tuberolachnini and the other Lachninae used for this study were examined via a Leica DM 3000 LED light microscope and photographed via a Leica MC 190 HD camera. Final figure processing was performed via PhotoScape 3.7 (photoscape.org) and IrfanView 64 (irfanview.com). The following abbreviations are used: **FT I**-first segment of the fore tarsus; **MT I**-first segment of the middle tarsus; **HT I**-first segment of the hind tarsus.

### Scanning *electron* microscopy

Lachninae specimens used for antennal sensilla and tarsi sensilla analyses were preserved in 80% ethanol for several days. Dehydration was accomplished through an ethanol series of 80%, 90%, and 96% and two changes of absolute ethanol for 10 min each. Absolute ethanol-dehydrated samples were treated with chloroform for 24 h. Dehydrated and cleaned samples were dried using a Leica EM CPD 300 auto critical point dryer (Leica Microsystems, Vienna, Austria). Dry samples were mounted on aluminium stubs with double-sided adhesive carbon tape and sputter-coated with a 30 nm gold layer in a Quorum 150 T ES Plus sputter coater (Quorum Technologies Ltd., Laughton, East Sussex, UK). The samples were imaged with a Hitachi SU8010 field emission scanning electron microscope (Hitachi High-Technologies Corporation, Tokyo, Japan) at 5–10 kV accelerating voltage with a secondary electron detector (SED). Final figure processing was performed via PhotoScape 3.7 (photoscape.org) and IrfanView 64 (irfanview.com).

### Molecular analyses

#### Taxon sampling and identification

A total of 294 individuals of 125 species belonging to all five recognized tribes of Lachninae were included in the phylogenetic analyses (Additional file 1). We used taxa from previous studies [11, 29, 41, 48–53] with 11 additional species: *Lachnus sorini* Binazzi & Remaudière, 2006; *Miyalachnus sorini* Kanturski & Lee, 2024; *Miyalachnus* sp.; *Nippolachnus micromeli* (Shinji, 1924); *Nippolachnus* sp.; *Pseudessigella brachychaeta* Hille Ris Lambers, 1966; *Pyrolachnus* spp.; *Sinolachnus niitakayamensis* (Takahashi, 1925); *Sinolachnus* sp.; *Sinolachnus yushanensis* Kanturski, Lee & Yeh, 2022; and *Tuberolachnus* sp. 2. Species identification was performed by Mariusz Kanturski through detailed morphological examination. Twelve outgroup taxa (1 species of Adelgidae, 4 species of Greenideinae, 2 species of Hormaphidinae, 1 species of Phloeomyzinae, 1 species of Phylloxeridae, and 1 species of Tamaliinae) were selected on the basis of recent molecular phylogenetic reconstructions of aphids [8, 54]. The GenBank accession numbers of the downloaded sequences are listed in Additional file 1. The classification of Chen et al. [11] is used as a background classification, and all taxon names follow Favret [1]. All samples were collected directly into 70–95% ethanol and stored at -20 °C for DNA extraction.

#### DNA extraction, PCR, sequencing, and alignment

Whole-genome DNA was extracted from a single aphid specimen for each sample following the manufacturer’s protocol for the LaboPass Tissue Kit (COSMO Genentech Korea). All voucher samples were deposited in the Zoological Collection of the Faculty of Natural Sciences, University of Silesia in Katowice, Katowice, Poland (DZUS). We used four molecular markers, three mitochondrial markers, i.e., cytochrome oxidase c subunit I (*COI*), cytochrome oxidase c subunit II (*COII*), and cytochrome oxidase b (*CytB*), and one nuclear marker, i.e., elongation factor-1-α (*EF1 α*), which have been widely used in aphid phylogenetic studies [7, 11, 29, 55, 56]. The polymerase chain reaction (PCR) primer sets used in this study are listed in Additional file 2. PCR was conducted using AccuPower PCR PreMix (Bioneer Corp., Daejeon, Korea) in 20 μl reaction mixtures under the following conditions: initial denaturation at 94 °C for 3 min, followed by 35 cycles at 94 °C for 30 s, an annealing temperature of 45–53 °C for 30–60 s, an extension at 65–72 °C for 60–90 s, and a final extension at 72 °C for 5–10 min. All PCR products were evaluated using 1.5% agarose gel electrophoresis. Successfully amplified samples were purified and sequenced directly using an automated sequencer (ABI PrismH 3730XL DNA Analyser) at Macrogen, Inc. (Korea).

The derived forward and reverse chromatograms were initially assembled and examined via SeqMan Pro ver. 7.1.0 (DNA Star, Inc., Madison, Wisconsin, USA) for analysis. Poor-quality sequences were excluded to minimize errors and confusion. To expand taxon diversity, we also utilized GenBank references for the four markers *COI*, *COII*, *CytB*, and *EF1α* (Additional file 1). Each dataset was aligned with the online utility MAFFT ver. 7 [57, 58], and uncertain anterior and posterior regions were removed as 1,227 bp (*COI*), 668 bp (*COII*), 730 bp (*CytB*), and 777 bp (*EF1α*). For *EF1α*, the intron regions were removed prior to alignment. Before the analyses, we confirmed that the sequences could be properly translated to protein sequences using Editseq (DNA star, Inc.). In each dataset, no pseudogenes or heteroplasmy that would cause misleading results were found [59, 60]. The aligned sequence data were combined with Sequence Matrix window ver. 1.7.8 [61].

### Phylogenetic analyses

Phylogenetic inference was conducted for a total of 3402 bp of the combined dataset of four concatenated genes via maximum likelihood (ML) and Bayesian inference (BI) methods.

For BI analysis, the best-fitting nucleotide substitution model for each gene was selected using IQ-Tree [62]. The GTR + I + Г, GTR + Г, and GTR + I models were selected for 12 subsets (Additional file 3). BI analyses were performed using MrBayes ver. 3.2.6 [63]. For the analyses, four chains (three heated chains and one cold chain) were run, starting from a random tree and proceeding for 10 million Markov chain Monte Carlo (MCMC) generations, sampling chains every 100 cycles. To ensure that the distribution had stabilized, Tracer ver. 1.4.1 [64] was used to view the graphical representation of MCMC chain mixing. In total, 137,733 trees were sampled, and the first 25% of the trees were discarded as burn-in. A 50% majority-rule consensus tree was constructed from the remaining trees to estimate posterior probabilities.

ML analysis was conducted using IQ-Tree v.2.3.6 following the 12-partition scheme. According to a comparative study, this program usually achieves trees with higher likelihood values than other programs do [65]. For the analyses, the best-fit substitution model was automatically determined for each partition using the Bayesian information criterion (BIC), as implemented in IQ-Tree v.1.6.5 [65]. The significance of the inferred relationship was assessed by an ultrafast bootstrapping (UFB) with 1000 replications. To view and place colours on branches and node values in the trees, Figtree v1.3.1 [66] was used.

## Results

### Phylogenetic relationships

**Fig. 3:**
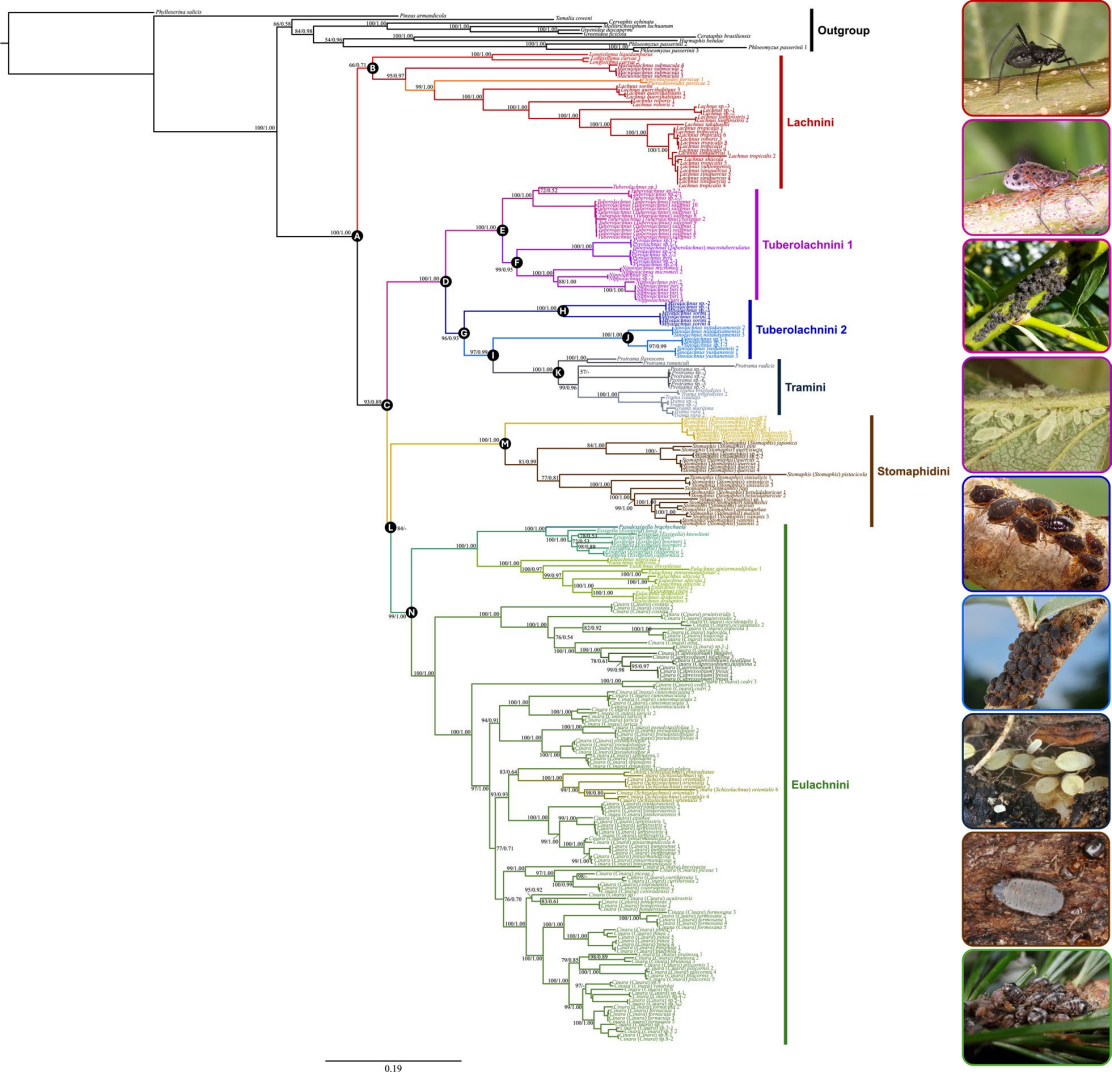
Phylogenetic relationships of the Lachninae from the combined dataset of 3,402 bp. Summarized results of the maximum likelihood (ML) tree in IQ-TREE. The two numbers near the nodes represent the posterior probabilities (PP) from the Bayesian inference (BI) and the ultrafast bootstrap (UFB) support values from the maximum likelihood result

Our phylogenetic analyses using Bayesian inference (BI) (Additional file 4) and maximum likelihood (ML) methods on the combined dataset yielded similar topologies, which we have summarized visually in Fig. 3 using the consensus ML tree. These results partially align with recent phylogenetic hypotheses, such as those proposed by Chen et al. [11]. We recovered strong support for monophyletic Lachninae (Fig. 3, node A, PP: 1.00, UFB: 100). Lachninae consisted of two main clades: one comprising Lachnini (Fig. 3, node B) and the other encompassing the remaining tribes (Fig. 3, node C). The phylogenetic position of Stomaphidini exhibited low resolution in the BI tree and, contrary to the findings of Chen et al. [11], formed a sister group with Eulachnini (Fig. 3, node L). Four tribes, Lachnini, Tramini, Stomaphidini, and Eulachnini were consistently recovered as monophyletic (Fig. 3, nodes B, K, M, and N). However, our analysis revealed that Tuberolachnini is not monophyletic. Tuberolachnini was found to be paraphyletic (Fig. 3, nodes E, H, J). Interestingly, the *Miyalachnus* (Fig. 3, node H, PP: 1.00, UFB: 100) and *Sinolachnus* (Fig. 3, node J, PP: 1.00, UFB: 100) clades were distinctly separated from the core members of Tuberolachnini (*Tuberolachnus* + *Pyrolachnus* + *Nippolachnus*) with perfect support (Fig. 3, node E, PP: 1.00, UFB: 100). *Pyrolachnus* and *Nippolachnus* formed a sister relationship (Fig. 3, node F, PP: 0.95, UFB: 99). Our analysis also revealed that Tramini formed a sister relationship with the *Sinolachnus* clade (Fig. 3, node I, PP: 0.99, UFB: 97; Fig. 4). Notably, *Miyalachnus* species (Fig. 3, node H), previously classified within the genus *Pyrolachnus*, formed a sister clade to the *Sinolachnus* + Tramini clade (Fig. 3, node G, PP: 0.93, UFB: 96; Fig. 4).

**Fig. 4:**
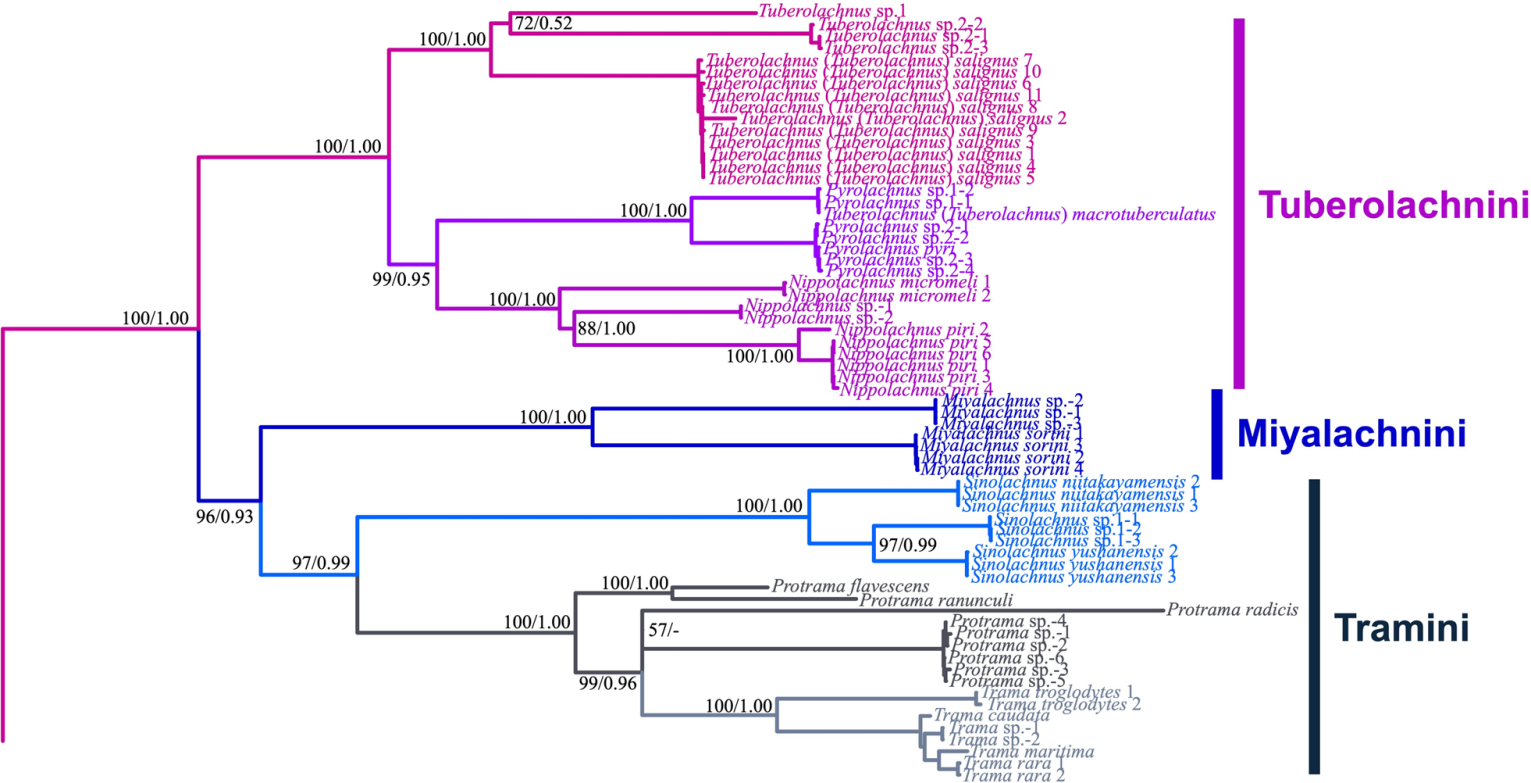
Part of the Lachninae phylogenetic tree showing Tuberolachnini and allied tribes

### Taxonomic implications

Tribe Miyalachnini **trib**. **nov**. Kanturski & Lee Y.

Type genus *Miyalachnus* Kanturski & Lee Y, 2024 [46]: 4

***Diagnosis***. Members of this new tribe within Lachninae are characterized by the following set of features is used:

1. Numerous sense pegs (13–21) on the first segments of tarsi, especially on the first segment of the fore tarsi (in all remaining tribes, the first segments of tarsi bear at most 9 sense pegs);
2. One accessory sensillum on the last antennal segment is moved to the PT (in all remaining tribes, in addition to the genus *Sinolachnus*, accessory rhinaria are all on the BASE or all of them are moved to the PT, as in the genus *Nippolachnus*);
3. Dorsal side of body with head covered in small denticles (in all remaining tribes, the species are characterized by a smooth or only wrinkled dorsum, except *Sinolachnus*, in which only the thorax and abdomen are characterized by denticles on the dorsal side);
4. Alate viviparous females may be distinguished by uniformly brown wings and blunt tip of pterostigma (without such a combination of characters in the other tribes).

**Etymology**: The name of the new tribe is derived from its type genus, *Miyalachnus*.

### Revised Lachninae tribal classification

#### Tribe Eulachnini Baker, 1920

**Fig. 5:**
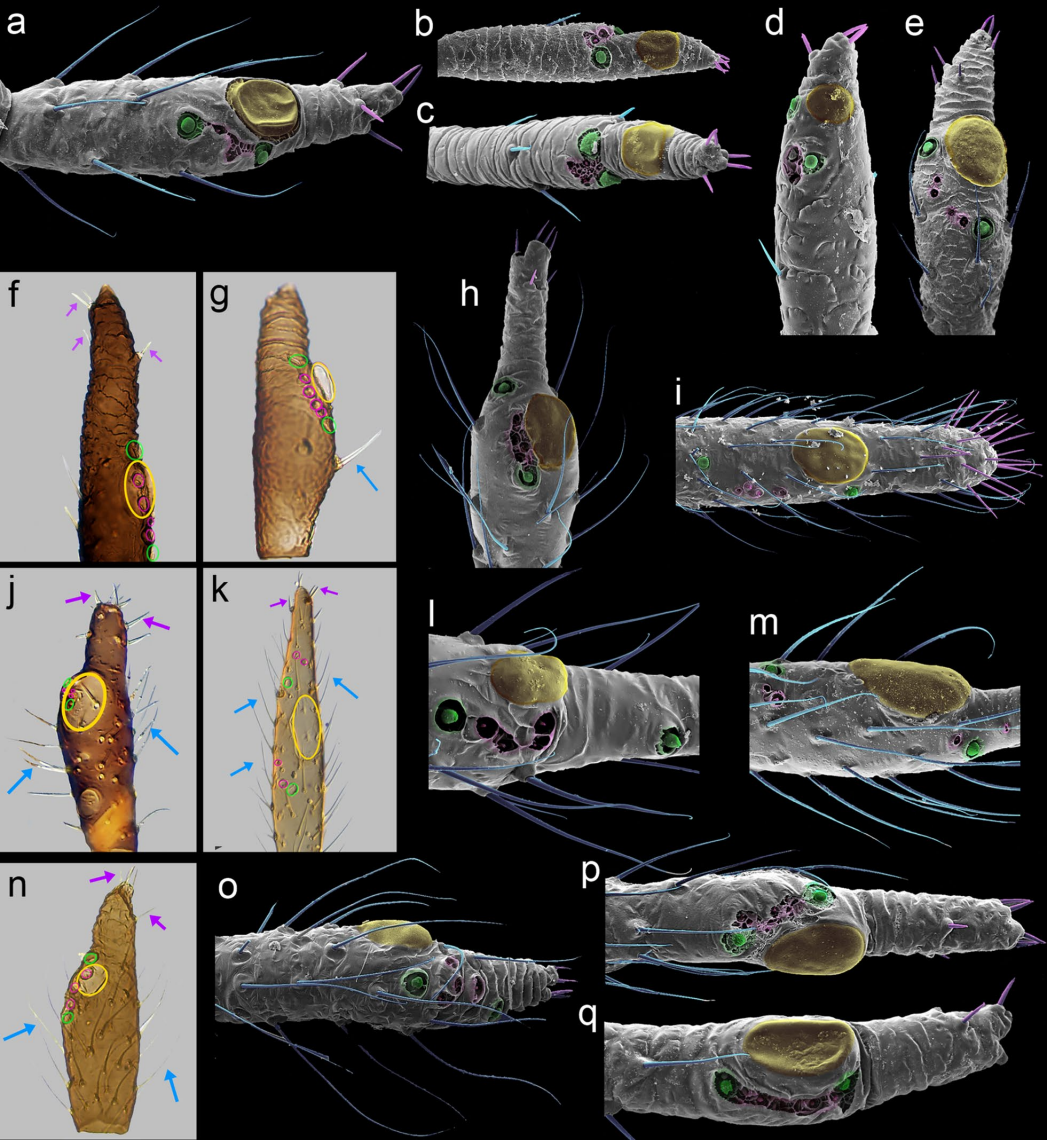
Lachninae representatives last antennal segment sensilla arrangement: Eulachnini: (**a**) *Cinara*, (**b**) *Essigella*, (**c**) *Eulachnus*, (**d**) *Pseudessigella*; Lachnini: (**e**) *Lachnus*, (**f**) *Longistigma*, (**g**) *Pterochloroides*; Miyalachnini: (**h**) *Miyalachnus*; Stomaphidini: (**i**) *Stomaphis*; Tramini: (**j**) *Eotrama*, (**k**) *Protrama*, (**l**) *Sinolachnus*, (**m**) *Trama*; Tuberolachnini: (**n**) *Indolachnus*, (**o**) *Nippolachnus*, (**p**) *Pyrolachnus*, (**q**) *Tuberolachnus*. Type I trichoid sensilla (blue), Type II trichoid sensilla (purple), large multiporous placoid sensilla-major rhinaria (yellow), small multiporous placoid sensilla-accessory rhinaria (green), and sunken coeloconic sensilla-accessory rhinaria (pink)

**Fig. 6:**
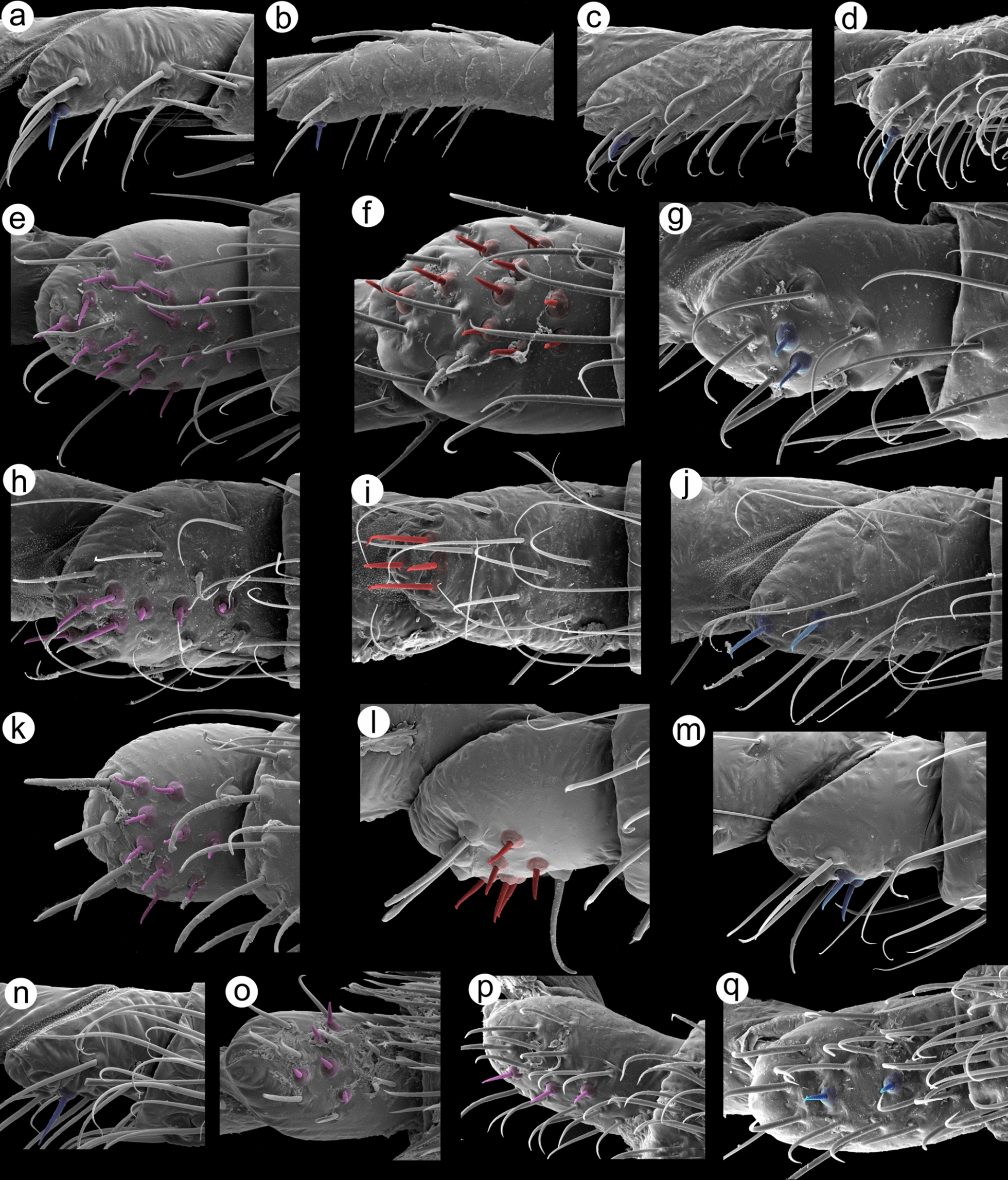
Lachninae representatives first segments of tarsi peg-like sensilla: Eulachnini: (**a**) HT I of *Cinara*, (**b**) HT I of *Essigella*; Lachnini: (**c**) HT I of *Lachnus*, (**d**) HT I of *Maculolachnus*; Miyalachnini: (**e**) FT I of *Miyalachnus*, (**f**) MT I of *Miyalachnus*, (**g**) HT I of *Miyalachnus*; Stomaphidini: (**h**) FT I of *Stomaphis*, (**i**) MT I of *Stomaphis*, (**j**) FT I of *Stomaphis*; Tramini: (**k**) FT I of *Sinolachnus*, (**l**) MT I of *Sinolachnus*, (**m**) HT I of *Sinolachnus*; Tuberolachnini: (**n**) HT I of *Nippolachnus*, (**o**) FT I of *Pyrolachnus*, (**p**) FT I of *Tuberolachnus*, (**q**) HT I of *Tuberolachnus*; peg-like sensilla on FT I (purple), peg-like sensilla on MT I (red), peg-like sensilla on HT I (blue)

- Comprises *Cinara*, *Essigella*, *Eulachnus* and *Pseudessigella*,
- Associated with coniferous trees and shrubs on which they feed on branches and bark (*Cinara* except the species of the subgenus *Schizolachnus*) and needles (*Essigella*, *Eulachnus*, *Pseudessigella* and the *Cinara* subgenus *Schizolachnus*),
- Eulachnini can be distinguished from other Lachninae by the following set of characters: **1**) accessory rhinaria arranged in one group at the lateral side (*Cinara*) (Fig. 5a), under the major rhinarium (*Essigella*, *Eulachnus*) (Fig. 5b, c) or separated from one accessory rhinarium at the side, whereas the rest lie under the major rhinarium (*Pseudessigella*) (Fig. 5d); **2**) 1–1–1 sense pegs of tarsi; and **3**) the basal length of HT I is much shorter than the dorsal length (Fig. 6a, b).

#### Tribe Lachnini Herrich-Schaeffer, 1854

- Comprises *Lachnus*, *Longistigma*, *Maculolachnus* and *Pterochloroides*,
- Associated with trees and shrubs of different plant families (mostly Fagaceae and Rosaceae), where they feed mostly on branches,
- Members of Lachnini can be distinguished from other Lachninae by the following set of characters: **1**) primary rhinaria on ANT V and VI (large multiporous sensilla) always with a sclerotic collar or rosette on the edge and accessory rhinaria on the last antennal segment in a crescent-shaped line (Fig. 5e-g), **2**) 1–1–1 sense pegs on tarsi (Fig. 6c, d); if there are more sense pegs, then in apterous viviparous females, the abdomen with large marginal plates and alate viviparous females with extremely long and fine pterostigma end (*Longistigma*).

#### Tribe Miyalachnini Kanturski & Lee

- Comprises *Miyalachnus*
- Associated with *Prunus* and *Cerasus*, which often feed on young branches or suckers and are often covered by soil shelters built by ants,
- They can be distinguished from the other Lachninae tribes by the following set of characters: **1**) Accessory rhinaria of all morphs with one (small multiporous placoid sensillum) moved to the PT and the rest of them on the lateral side of the major rhinarium (Fig. 5h), **2**) apterous viviparous females with the largest number of sense pegs of tarsi (Fig. 6e-g) and dorsal side of the abdomen with well-developed denticles, and **3**) alate viviparous females with uniformly brown wings.

#### Tribe Stomaphidini Mordvilko, 1914

- Comprises *Stomaphis*,
- Associated with different trees from Fagaceae, Salicaceae, Aceraceae and *Pinus* (in Asia), where they feed on trunks,
- Compared with other Lachninae members, Stomaphidini can be distinguished by the following set of characters: **1**) the labium (rostrum), which is much longer than the body; **2**) the accessory rhinaria lie in line far from each other but only on the basal part of the segment (Fig. 5i); and **3**) the sense pegs of tarsi are longer than half of the length of the rest of the setae and approximately [(5–8)—(4–5)—2] (Fig. 6h-j).

#### Tribe Tramini Herrich-Schaeffer, 1854

- Comprises *Eotrama*, *Protrama*, *Sinolachnus* and *Trama*,
- Associated with trees and shrubs of Elaeagnaceae and Rosaceae (two species are associated with *Rubus* spp.), where they feed on branches (*Sinolachnus*), and with different herbaceous plants mostly of the family Asteraceae, where they feed on roots (*Eotrama*, *Protrama*, *Trama*),
- They can be distinguished from other Lachninae tribes by the following set of characters: **1**) accessory rhinaria lie singly and far from each other, but some of them are also located on the PT (Fig. 5k) (then also always HT II extremely long, longer than half of the tibiae); **2**) accessory rhinaria lie all in a group at the side of the major rhinarium (Fig. 5j), or one is moved to the PT (Fig. 5l) (then, HT II never longer than half of the length of the tibiae); and **3**) tarsi with 5–3–2 or [(6–8)—(4–5)—2] sense pegs (Fig. 6k-m).

#### Tribe Tuberolachnini Oestlund, 1942

- Comprises *Indolachnus*, *Nippolachnus*, *Pyrolachnus* and *Tuberolachnus*,
- Associated with Salicaceae (*Tuberolachnus*) and woody Rosaceae, which may feed on branches (*Pyrolachnus*) or leaves and young shoots (*Indolachnus*, *Nippolachnus*),
- They may be distinguished from other Lachninae by the following characteristics: **1**) accessory rhinaria all in a group on the side of the major rhinarium (*Pyrolachnus* and *Tuberolachnus*) (Fig. 5p, q) and then more than 1–1–1 sense pegs on tarsi (Fig. 6o-q) or all accessory rhinaria (sometimes one not) moved to the PT (Fig. 5n, o) and then tarsi with 1–1–1 sense pegs (*Indolachnus* and *Nippolachnus*) (Fig. 6n), **2**) alate viviparous females with hyaline wings (*Tuberolachnus salignus*, *Indolachnus himalayensis* and *Nippolachnus*) or with only the basal part pigmented but then the pterostigma of normal length (*Pyrolachnus*).

## Discussion

In recent years, Lachninae has been the subject of numerous analyses and studies, largely reflecting the focus and specialization of the first author, referring to Lachninae morphology [23, 67–70], taxonomy and systematics [16, 19, 23, 38, 45, 71–81], biodiversity and biology [82–85] and phylogeny [16, 45, 76]. Our reconstruction of Lachninae resulted in a comprehensive and well-resolved phylogenetic tree, incorporating the most diverse array of taxa to date. While Lachninae has been the subject of several phylogenetic and evolutionary studies [11, 29, 49, 51], the tribe Tuberolachnini has been underrepresented in previous phylogenies, with a few species included [11, 29]. The classification of Tuberolachnini has been particularly contentious, leading to ongoing taxonomic debates. Only very recently has a more stable classification system been proposed [1]. Notably, our study is the first to include *Miyalachnus* and *Sinolachnus* in a phylogenetic analysis, addressing a significant gap in previous research.

### Relationships within Tuberolachnini

In the present study, our results clearly refute the monophyly of Tuberolachnini, primarily due to the nesting of Tramini (Fig. 3, node D). This finding aligns with longstanding questions about monophyletic Tuberolachnini, stemming from morphological similarities between some of its species and those of Tramini [23, 86]. Our phylogenetic analysis revealed that certain taxa previously considered part of Tuberolachnini, such as *Miyalachnus* and *Sinolachnus*, consistently grouped with Tramini rather than core Tuberolachnini members. Specifically, the *Miyalachnus* group formed a separate clade distinct from the tribe Tuberolachnini (Fig. 3, node H). The *Sinolachnus* group was associated with the Tramini clade rather than other Tuberolachnini species.

Tuberolachnini exhibits a wide range of morphological and biological diversity within Lachninae. We can distinguish two main groups: *Nippolachnus,* with the newly erected genus *Indolachnus*, and *Pyrolachnus,* with *Tuberolachnus*. *Nippolachnus* species are characterized by apterous viviparous females with narrow-oval, elongate body which is rather poorly pigmented and unsclerotized. Furthermore, they are characterized by relatively short ANT III compared with other segments (IV + V or IV + V + VI) and an arrangement of accessory rhinaria (two small placoid sensilla and sunken coeloconic sensilla), which very often are all located on the terminal process. *Nippolachnus* alate viviparous females also present many features that are specific to themselves. These are relatively short antennae with extremely large secondary rhinaria (small multiporous placoid sensilla), one of the largest types in Aphididae. The second important characteristic is the pattern of sclerotization of the abdomen, which, in the vast majority of species, is very complicated but is constant across species. Although *Indolachnus* is currently unavailable for molecular research, phylogenetic results based on the numerous morphological and biological characteristics described by Kanturski et al. [45] indicate that the newly erected genus is a sister group to the monophyletic *Nippolachnus*. In general, *Indolachnus* is similar to *Nippolachnus* but differs from the latter in the red pigmented body in alive specimens [87] and the dark pigmented legs in mounted specimens. Kanturski et al. [45] listed several differences between *Indolachnus* and the remaining *Nippolachnus* species, the most important of which are the differences between accessory rhinaria (two small multiporous placoid sensilla and sunken coeloconic sensilla) arrangement in relation to the major rhinarium (large multiporous placoid sensillum), mesosternal furca shape and hind femora sensilla characters. The alate viviparous females of *Indolachnus* are also characterized by a membranous abdomen and twice-branched media of forewings. Finally, *Nippolachnus* and *Indolachnus* members, in contrast to other Tuberolachnini and many Lachninae, feed on leaves (and additionally, in some cases, on young stems) and never on woody parts. This type of feeding place is unique throughout Lachninae, and only some representatives of Eulachnini (*Essigella*, *Eulachnus*, *Pseudessigella* and the *Cinara* subgenus *Schizolachnus*) can be considered similar, as they feed on the needles of conifers [3, 4]. *Nippolachnus* and *Indolachnus himalayensis* also share one characteristic and unique feature within Lachninae—the residual triommatidium, which is almost invisible (especially in mounted slide specimens) without an ocular tubercle and is hidden behind or under the compound eye. This character-or its “absence” (being invisible on mounted slides)-probably led to *Nippolachnus* being placed in its own tribe, Nippolachnini, in the past [88]. As Kanturski et al. [23] demonstrated the presence of triommatidia in *Nippolachnus*, it has been much easier to understand the presence of the latter in Tuberolachnini. Compared with *Nippolachnus* and *Indolachnus*, *Pyrolachnus* and *Tuberolachnus* are more similar to each other first because of the large, egg-shaped bodies of apterous viviparous females, which are additionally dark coloured as live specimens. Additionally, sclerotization is very often much more developed at least on the head and thorax (e.g., *Tuberolachnus salignus*, *T*. *macrotuberculatus* and *Pyrolachnus* species), to completely sclerotised (*T*. *scleratus*). Apterae of both genera have moreover more strongly sclerotized siphunculi, shorter ultimate rostral segments and much longer antennal segment III in relation to the length of the remaining segments than do those of *Nippolachnus* and *Indolachnus*. In apterous as well as in alate viviparous females of both genera, accessory sensilla on the last antennal segment are normally located on the ANT VI BASE, lying rather tightly and near the major rhinarium (Fig. 5p, q). In the case of the feeding place, members of both genera are similar to most Lachninae and can be found mostly on woody branches (in the case of *Tuberolachnus,* the branches can be green but then never young).

The long neglected but new, and definitely worth using, features in Lachninae that have additionally been shown to be important in various studies (both taxonomical and phylogenetic) are the number and length of the “sense pegs” (or peg-like setae) on the ventral side of the first segments of the fore, middle and hind tarsi as well as the last antennal sensilla arrangement. As a result of detailed analyses of this feature in Tuberolachnini and other Lachninae, the number, range and length of sense pegs are consistent across genera and tribes. In the case of Tuberolachnini, we can observe once again two separate groups. *Nippolachnus* and *Indolachnus* are once again the most distinctive, with one sense peg (1–1–1) on the first segments of the fore, middle and hind tarsi, whereas *Tuberolachnus* is characterized by [(2–3)-(2–3)-(2)] sense pegs and *Pyrolachnus* has [(4–6)-(4–6)-(3–4)] of these sensilla. In the background presented thus far, the genus *Sinolachnus* has differed from the remaining Tuberolachnini members in many morphological characteristics. The first one is the secondary rhinaria of alate viviparous females, which are extraordinary within Aphididae owing to their great number (several dozen to well over a hundred on one segment), they are very small and protuberant (also quite an uncommon feature in aphids), and in this character, they are more similar to those in *Eotrama* and *Protrama*. Additionally, the presence of secondary rhinaria on the antennae (often on the last segment) of the apterous morphs makes *Sinolachnus* closer to Tramini than to Tuberolachnini. In general, *Sinolachnus* can be clearly found to be one of the most difficult Lachninae genera, which may explain why, in some cases, species from this genus have been described in other genera, such as *Maculolachnus* or *Cinara*. Kanturski et al. [19], during the revision of the genus, noted several differences in *Sinolachnus* from Tuberolachnini and similarities with Tramini, transferring it to the last tribe. The obtained molecular results confirmed the hypothesis and detailed morphological analyses conducted by these authors regarding the identity of *Sinolachnus* [19]. Obviously, in the future, a collection of the unusual difficult-to-obtain *Eotrama* will definitely and finally solve the questions about the relationships within Tramini. There is a high likelihood that *Eotrama*, along with *Sinolachnus*, will form a sister clade to the remaining genera (*Protrama* and *Trama*), and then the restoration of the subtribal classification (with Eotramina and Tramina) proposed by Czylok [36] will be needed. On the other hand, *Eotrama* may be a sister clade to *Protrama* and *Trama*, and then the rest will be sister clade to *Sinolachnus*, which would require a different approach to the classification of this tribe; this is the first author's ongoing project.

### Miyalachnini and other Lachninae tribes

The molecular analysis revealed that *Miyalachnus sorini* (previously *Pyrolachnus imbricatus nipponicus*) and *Miyalachnus* sp. formed a sister clade to the combined *Sinolachnus* and Tramini clade (Fig. 3, node G). Notably, despite this phylogenetic relationship, *Miyalachnus* and *Sinolachnus* share some morphological and biological similarities. These shared characteristics help explain their close relationships and align with the findings of Kanturski et al. [45], where both genera were grouped into a single clade. First, what distinguishes apterous viviparous females of *Miyalachnus* not only from *Sinolachnus* but also from the remaining Tramini and whole Lachninae is the large (the largest within the subfamily) number of sense pegs on the first segments of the fore tarsi and middle tarsi (Fig. 6h-j). *Sinolachnus* is the runner in the case of this characteristic, and the tarsi of Tramini members also bear many of these structures (4–6 on the fore tarsi) (Fig. 6k‒m). In *Miyalachnus,* the situation is similar: as in *Sinolachnus,* there is an accessory rhinaria arrangement where one of the two small multiporous placoid sensilla is separated from the rest of the accessory rhinaria (the second small multiporous placoid sensillum and four sunken coeloconic sensilla) and lies on the terminal process. On the other hand, after careful examination of the sensilla features, additional differences can be observed. The one separated accessory rhinarium lies much further from the major rhinarium in *Sinolachnus* (evidently on the PT) (Fig. 5l), and in *Miyalachnus,* the separated accessory rhinarium lies close to the major one, directly on the very base of the PT (Fig. 5j). The differences in both sensilla also extend to the morphological features of small multiporous placoid sensilla, which in *Miyalachnus* are flat (as in many Lachninae), surrounded by a gentle flange, and the sunken coeloconic sensilla lie together in a group in a cavity with many reinforcements. In *Sinolachnus*, on the other hand, small multiporous placoid sensilla are rounded or even capitate with sharp surrounding edges, and the sunken coeloconic sensilla form two separate pairs and lie mainly singly with much less reinforcement. Moreover, in *Miyalachnus* and *Sinolachnus*, shifting of part of all accessory rhinaria in Lachninae (and most Aphidinae) is known only in other Tramini (*Trama* and *Protrama*) and *Nippolachnus* (Tuberolachnini). This characteristic may also explain the close relationship between the clade of *Miyalachnus* and Tramini and Tuberolachnini. In the case of alate viviparous females, differences between genera and tribes are more evident, in addition to the same differences and patterns of the last antennal segment sensilla and peg-like setae on tarsi, which focus on pigmentation and other characteristics of forewings, such as venation and pterostigma shape.

In our study, Lachnini is a sister group to remaining tribes. Apterous viviparous females of members of this tribe exhibit remarkable similarity to each other (especially *Lachnus* and *Maculolachnus*, which on the other hand is very similar to *Sinolachnus*), with *Pterochloroides* and *Longistigma* having their own individual characteristics—dorsal spinal tubercles in the first case and marginal sclerotic plates on the abdomen in the latter. Despite the generic differences, all Lachnini can be characterized by only one sense peg on tarsi of all legs, with one exception in *Longistigma*, but additionally, all Lachnini members share one additional feature of antennal sensilla—an evident sclerotic rosette on the primary rhinaria (large multiporous placoid sensilla on antennae V and VI), which is absent in all remaining tribes (this feature may support the understanding of the separateness from other tribes in the molecular results). Our results revealed that, in the case of deciduous-feeding lachnids, Stomaphidini formed a sister group to Eulachnini, which can be explained first of all by the ability of some *Stomaphis* species which can feed on conifer trees and shrubs, like *S*. *abieticola* Sorin, 2012, *S*. *cupressi* (Pintera, 1965), *S*. *pini* Takahashi, 1920. The remaining characters which support the separateness of Stomaphidini from other Lachninae can be for example their exceptionally long rostrum (labium), which is the longest among Aphididae, and their large body size, which is also the largest among aphids, is the very poorly separated terminal process of the last antennal segment and numerous apical setae (type II trichoid sensilla) (Fig. 5h). In the case of *Stomaphis*, its biological features may also explain its position in contrast to the deciduous Lachninae clades, especially their feeding location, ant-driven speciation [14] and host alternation [89], although seasonal host alternation and heteroecy was also discovered in the case of the holocyclic *Nippolachnus piri* [45].

Our results allowed us to propose a new tribal classification of Lachninae based on six tribes, with a new one described as Miyalachnini. This proposal may, of course, raise questions why we decided to propose a new tribe instead of treating the one (on the other hand, strongly supported monophyletic) group formed by Tuberolachnini and Tramini as one tribe divided into subtribes. This will result in a four-tribe division system of the subfamily and may be similar to treating Eulachnini as one tribe. Taking into account the molecular phylogeny results, supported by detailed and intensive morphological studies, which in the case of Lachninae has been provided for the first time, our six-tribe classification seems to be more natural, especially considering the morphological characters of genera belonging to each group. The differences mentioned above between particular tribes support our proposal, especially due to a very high level of divergences between Tuberolachnini, Tramini and the newly established Miyalachnini in contrast to e.g., Eulachnini. Eulachnini are characterised by two main morphological groups of aphids-broad or egg-shaped *Cinara* (including all subgenera) and the spindle-shaped *Essigella*, *Eulachnus* and *Pseudessigella*. All other characters including only one sense-peg, relatively long HT I are similar, even the characters of sensilla on the last antennal segment (all sensilla, even separated in *Pseudessigella* are distributed only on the basal part of the segment). On the other hand Miyalachnini, Tramini and Tuberolachnini are groups of much more diverse and complex genera considering the same characters. Genera belonging to Tuberolachnini also form two separate morphological groups: the spindle-shaped *Nippolachnus* and the much broader *Pyrolachnus*, *Tuberolachnus* and *Indolachnus*, despite not being included in the molecular analyses. Tuberolachnini defined in this study are characterised by quite different last antennal sensilla characters which in *Nippolachnus* are almost completely moved to the terminal process while in the other genera they are rather together and lie near the major rhinarium on the basal part. They are moreover different in the number of sense-pegs on tarsi, wings pigmentation (only in *Pyrolachnus* bases pigmented), and moreover as the only group characterised by the presence of a single, large, evident and sclerotised dorsal tubercle on the abdomen. The mentioned sets of individual characters and differences support the proposed classification of Lachninae and correspond with the clades formed on the basis of the molecular studies.Comparing molecular studies with considering the morphological differences and similarities is close to our current knowledge of Lachninae, taking into account morphology, biology and ecological observations.

However, we are convinced that more detailed analyses, taking into account not only the current results but also data from known fossils in a total-evidence approach, will help to much better understand the complexity of Lachninae in the future. This, of course, needs studies of the molecular phylogenetic statuses of *Eotrama* and *Indolachnus*, which can uphold or change this Lachninae classification, which is the first author's ongoing research.

## Conclusions

A new tribe within Lachninae was described together with comparative diagnoses of all remaining tribes. We provided the first analyses of the antennal sensilla arrangement and tarsal peg-like sensilla number to show the similarities and differences among the groups, which have also been confirmed via molecular phylogeny. This resolves a long-standing issue, with the identity and phylogenetic position of some poorly known genera treated earlier as members of Tuberolachnini, and justifies the continuation of further and deeper analyses of the phylogeny and relationships within Tramini.

## Supplementary Information

**Additional file 1:** Aphids samples used for the study.

**Additional file 2:** Primers used in this study.

**Additional file 3:** Selected subsets for BI analysis.

**Additional file 4:** Phylogenetic relationships of the Lachninae from the combined dataset of the Bayesian Inference (BI).

## Data Availability

All the data generated or analysed during this study are included in this published article and its supplementary information files.
