# Supplementary material for: Phylogenetic reconstruction of Tuberolachnini and Lachninae (Insecta, Hemiptera): Morphological and molecular analyses revealed a new tribe
Source: Front Zool. 2024 Nov 19;21:29. doi: 10.1186/s12983-024-00550-2 (PMC11575128; doi:10.1186/s12983-024-00550-2)
Supplement: Supplementary file 2 — Additional file 2: Primers used in this study. [file 12983_2024_550_MOESM2_ESM.pdf]

Phylogenetic reconstruction of Tuberolachnini and Lachninae (Insecta, Hemiptera): Morphological and molecular analyses revealed a new tribe

MARIUSZ KANTURSKI [1 *], YERIM LEE [2], HYOJOONG KIM [2]

[1] *Institute of Biology, Biotechnology and Environmental Protection, Faculty of Natural Sciences, University of Silesia in Katowice, Bankowa 9, 40-007 Katowice, Poland;* ***Corresponding author****:* Mariusz Kanturski, e-mail: mariusz.kanturski@us.edu.pl

[2] *Department of Biological Sciences, Kunsan National University, 558 Daehak-ro, Naun 2(i)-dong, Gunsan-si, Jeollabuk-do, Republic of Korea*

Supplementary file 2: Primers used in this study

Gene	Primer	Sequence	Annealing temperature	Product Size (bp)	Reference
COI	LCO1490	GGTCAACAAATCATAAAGATATTGG	45°C	658	Folmer et al., 1994
	HCO2198	TAAACTTCAGGGTGACCAAAAAATCA			
COII	2993	CATTCATATTCAGAATTACC	46°C	531	Stern, 1994
	A3772	GAGACCATTACTTGCTTTCAGTCATCT			
CytB	CP1	GATGATGAAATTTTGGATC	48°C	800	Harry et al., 1998
	CP2	CTAATGCAATAACTCCTCC			
	CB2	ATTACACCTCCTAATTTATTAGGAAT			
EF1α	EF3	GAACGTGAACGTGGTATCAC	53°C	831	von Dohlen et al., 2002
	EF2	ATGTGAGCAGTGTGGCAATCCAA			Palumbi, 1996
