# Supplementary figures and images for: Phylogenetic reconstruction of Tuberolachnini and Lachninae (Insecta, Hemiptera): Morphological and molecular analyses revealed a new tribe
Source: Front Zool. 2024 Nov 19;21:29. doi: 10.1186/s12983-024-00550-2 (PMC11575128; doi:10.1186/s12983-024-00550-2)

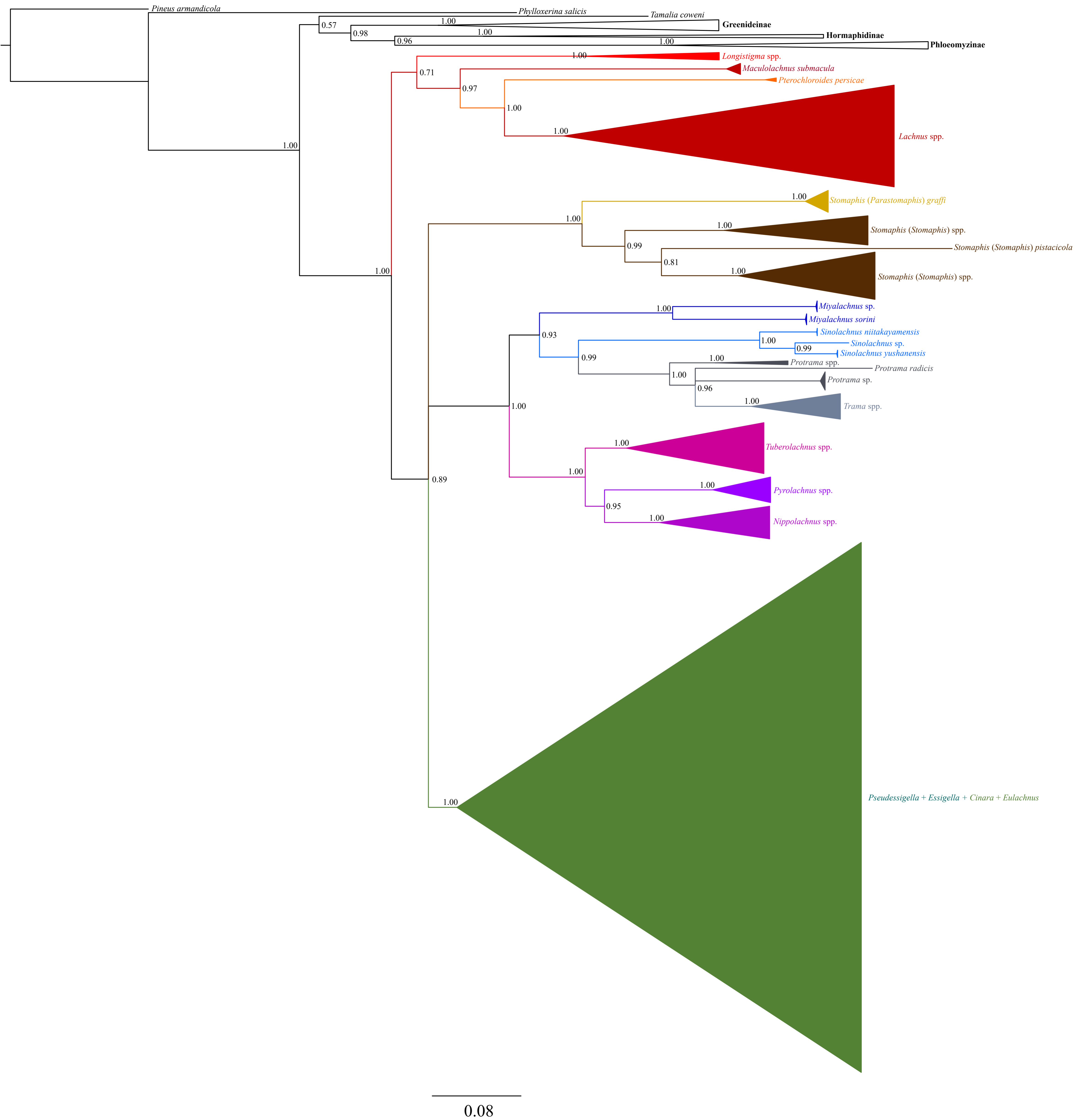

Supplement: Supplementary file 4 — Additional file 4: Phylogenetic relationships of the Lachninae from the combined dataset of the Bayesian Inference (BI). [file 12983_2024_550_MOESM4_ESM.pdf]
